# Whole-Genome Sequences of Two *Kazachstania barnettii* Strains Isolated from Anthropic Environments

**DOI:** 10.1093/gbe/evac007

**Published:** 2022-02-01

**Authors:** Hugo Devillers, Véronique Sarilar, Cécile Grondin, Lieven Sterck, Diego Segond, Noémie Jacques, Delphine Sicard, Serge Casaregola, Colin Tinsley

**Affiliations:** 1 SPO, Univ Montpellier, INRAE, Institut Agro, Montpellier, France; 2 Université Paris-Saclay, INRAE, AgroParisTech, Micalis Institute, Jouy-en-Josas, France; 3 French Armed Forces Biomedical Research Institute (IRBA), Department of Platforms and Technology Research, Molecular Biology Unit, Brétigny-sur-Orge, France; 4 Ghent University, Department of Plant Biotechnology and Bioinformatics, Ghent, Belgium; 5 VIB Center for Plant Systems Biology, Ghent, Belgium; 6 Université Paris-Saclay, INRAE, UMR BIOGER, Thiverval-Grignon, France

**Keywords:** whole-genome sequencing, sourdough bread, comparative genomics

## Abstract

Recent studies have suggested that species of the *Kazachstania* genus may be interesting models of yeast domestication. Among these, *Kazachstania barnettii* has been isolated from various microbially transformed foodstuffs such as sourdough bread and kefir. In the present work, we sequence, assemble, and annotate the complete genomes of two *K. barnettii* strains: CLIB 433, being one of the two reference strains for *K. barnettii* that was isolated as a spoilage organism in soft drink, and CLIB 1767, recently isolated from artisan bread-making sourdough. Both assemblies are of high quality with N50 statistics greater than 1.3 Mb and BUSCO score greater than 99%. An extensive comparison of the two obtained genomes revealed very few differences between the two *K. barnettii* strains, considering both genome structure and gene content. The proposed genome assemblies will constitute valuable references for future comparative genomic, population genomic, or transcriptomic studies of the *K. barnettii* species.

Significance

The *Kazachstania* genus contains more than 40 distinct species, isolated from a wide range of environments. Recent studies have demonstrated their predominance in several anthropic environments such as fermented food, highlighting their usefulness in the study of yeast domestication. Understanding the evolution of species requires reference-level genomes, but only a few high-quality genome assemblies and annotations are available for *Kazachstania* species. In this work, we propose the first genome sequences of two *K. barnettii* strains, CLIB 433 isolated from soft drink and CLIB 1767, isolated from wheat sourdough. The high-quality assemblies (N50 > 1.3 Mb) and annotations (BUSCO>99%) constitute a solid base to study the adaptation and evolution of this species.

## Introduction

Interest in the study of *Kazachstania* has grown in recent years, especially because *Kazachstania* species have been isolated from a wide range of environments of prime interest (Kabisch et al. 2016; Urubschurov et al. 2018; Faherty et al. 2019; García-Béjar et al. 2020; Morio et al. 2020; Wang et al. 2020; Mercier et al. 2021). Isolated from both wild and domesticated environments, they represent promising models to study domestication (Carbonetto et al. 2018).

Studying evolution and adaptation of an organism is very dependent on the availability of reference genomes as gold standards for the DNA sequences and gene annotations of the species. Such genomes are expected to have a high-quality assembly (i.e., the number of contigs/scaffolds should be close to the number of chromosomes) and a complete gene annotation. The *Kazachstania* genus contains more than 40 described species and it is one of the most closely related to the well-studied *Saccharomyces*. However, only three high-quality genome assemblies and annotations have been published to date: *Kazachstaniaafricana*, *Kazachstanianaganishii* (Gordon et al. 2011), and *Kazachstaniasaulgeensis* (Sarilar et al. 2017). Annotated genomes exist for two further species, *Kazachstaniaunispora* and *Kazachstaniaexigua*, but their assemblies remain highly fragmented (BioProject: PRJNA435582). Lastly, 17 different *Kazachstania* species have a draft assembly without annotation data (Shen et al. 2018; Faherty et al. 2019; Morio et al. 2020). The *Kazachstania* genus, together with others such as *Saccharomyces*, *Nakaseomyces*, and *Tetrapisispora*, belongs to the branch of *Saccharomycetaceae* that underwent a whole-genome duplication (WGD) event, followed by massive differential gene loss, which generated an important interspecific diversity (Morel et al. 2015), as well as a variable number of duplicated genes, known as ohnologs (Wolfe and Shields 1997; Kellis et al. 2004).

In this work, we provide the first reference genomes of *Kazachstania barnettii*, formerly *Saccharomyces barnettii* (Vaughan-Martini 1995; Kurtzman 2003), a species that has been isolated predominantly from food substrates, such as kimchi (lacto-fermented vegetables), sauerkraut, soft drink, and sourdough bread starter (Vaughan-Martini 1995; Urien et al. 2019; Kim et al. 2020). Two different strains are sequenced in this work: firstly, CLIB 433, similar to the CBS 6946 that is one of the two strains used to describe the species (Vaughan-Martini 1995) and was isolated from soft drink; secondly, CLIB 1767 that was isolated from a French wheat sourdough (Urien et al. 2019). Here, we present the characteristics of the two assemblies, and undertake a comprehensive comparison of the two *K. barnettii* genomes.

## Results and Discussion

### Assembly and Annotation Overview

*Kazachstania barnettii* is a post-WGD species and the expected number of chromosomes in such organisms generally ranges between 12 and 16. The obtained assemblies for the two *K. barnettii* strains CLIB 433 and CLIB 1767 comprise 14 and 15 scaffolds, respectively. Basic statistics of the genome assemblies and annotations of the two strains are presented in table 1. With a similar overall length of 12.6 Mb and G + C contents of about 33.5%, these two assemblies have an N50 statistic greater than 1.3 Mb with an L50 equal to 4. Note that the proposed assemblies do not include the mitochondrial genome.

**Table 1:** Assembly and Annotation Statistics of the Two *Kazachstania barnettii* Strains CLIB 433 (Reference) and CLIB 1767 (Sourdough) and of the *Kazachstania saulgeensis* Strain CLIB 1764 (Sourdough)

	CLIB 433	CLIB 1767	CLIB 1764
Scaffold count^a^	15	14	17
Overall size^b^	12,610,268	12,616,033	12,935,755
Scaffold max. length	2,518,272	1,873,880	2,959,652
Scaffold min. length	16,307	123,979	17,310
Average G + C content (%)	33.51	33.46	32.19
N50/L50	1,360,346/4	1,404,614/4	1,371,409/4
Assembly gap count	89	94	77
CDS count (pseudo)	5,322 (48)	5,316 (49)	5,376 (67)
Intron count	193	193	199
tRNA count	195	199	196
BUSCO^c^ score (%)	C: 99.3 (S: 97.6, D: 1.7), F: 0.1, M: 0.6	C: 99.2 (S: 97.5, D: 1.7), F: 0.1, M: 0.7	C: 98.9 (S: 97.2, D: 1.7), F: 0.1, M: 1.0

Note.—C, complete copy; S, single copy; D, duplicated copy; F, fragmented copy; M, missing.

a The cutoff threshold for definition of a scaffold was 10 kb.

b Genome size and other lengths are given in bases.

c BUSCO version 4.0.5, based on the saccharomycetes_odb10 data set (*n* = 2,137 proteins).

The genome annotation of the strain CLIB 433 mainly consists of 5,322 protein-coding genes, including 48 pseudogenes or incomplete genes (e.g., interrupted by an assembly gap). Annotation of the strain CLIB 1767 gave 5,316 protein-coding genes, including 49 pseudogenes or incomplete genes. Both annotations have 188 intron-containing genes (183 with 1 intron, 5 having 2 introns). In addition, CLIB 433 also contains 18 Ty retrotransposon elements (including 16 relics) and 195 tRNA loci while the CLIB 1767 has 13 Ty retrotransposon elements (12 relics) and 199 tRNA loci. Finally, the rRNA repeats (two units are manually assembled and annotated), are located on the fifth scaffolds of both strains.

Completeness of the two assemblies was evaluated with BUSCO considering the Saccharomycetes data set (2,137 proteins). Obtained BUSCO scores were 99.3% and 99.2% for CLIB 433 and CLIB 1767, respectively (see details in table 1). By comparison, the *K. saulgeensis* CLIB 1764 assembly had a BUSCO score of 98.9% (table 1).

### Comparison of the Two *K. barnettii* Genomes

As a first stage in comparison of these strains, the assembled sequences from the two *K. barnettii* strains CLIB 433 and CLIB 1767 were compared with identify possible chromosomal rearrangements and segmental duplications or losses. The complete genome of *K. saulgeensis* strain CLIB 1764 was considered as a reference outgroup because it is both a closely related species and has been isolated from bread sourdough starter as was *K. barnettii* strain CLIB 1767.

**Fig. 1.**
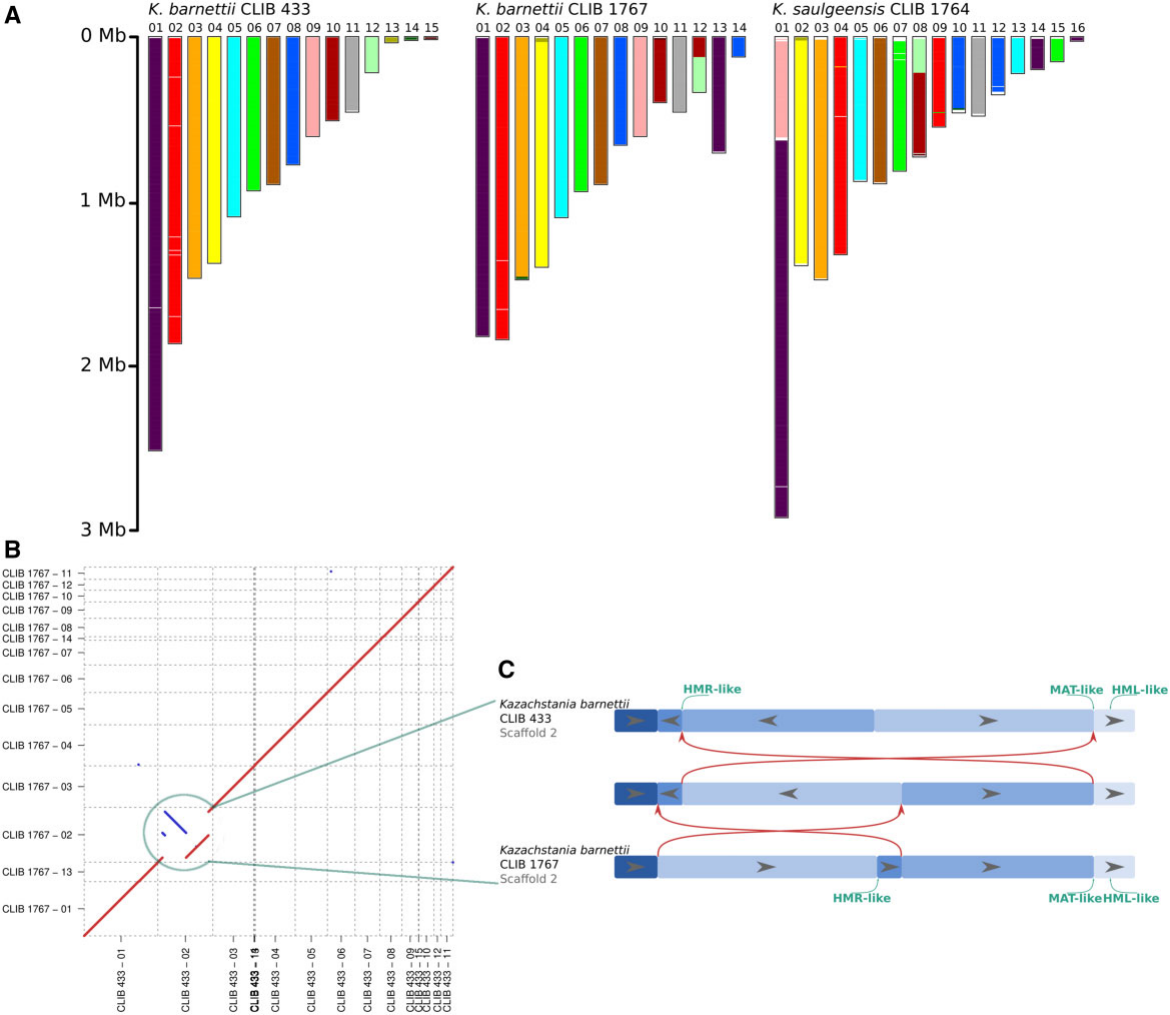
(*A*) Synteny blocks between the *Kazachstania barnettii* strains CLIB 433 (reference) and CLIB 1767 (sourdough) and the *Kazachstania saulgeensis* strain CLIB 1764 (sourdough). Each synteny block is colored according to the scaffolds of CLIB 433. Thus, for example, blocks from the scaffold 01 in CLIB 433 (in violet) are found in scaffolds 01 and 13 in the assembly of CLIB 1767 and in scaffolds 01, 14, and 16 in the assembly of *K. saulgeensis* CLIB 1764. (*B*) Dot plot between the two *K. barnettii* strains, CLIB 433 (reference) and CLIB 1767 (sourdough) based on MUMmer matches. To facilitate reading and interpretation of, scaffolds are reordered in accordance with the synteny block organization in (*A*). (*C*) Comparison of the structure of the scaffolds of the two *K. barnettii* assemblies. Red arrows represent possible inversion events between the two scaffolds in one possible sequence leading from one strain’s organization to the other. The central band represents the corresponding intermediate organization. Positions of mating type loci are indicated in green.

Conservation of gene order was investigated between the three assemblies, using SynChro, from the CHROnicle tool suite. A minimum of four genes were required to define a synteny block. The conserved synteny blocks are shown in figure 1*A*, which reveals a very high level of synteny between the two *K. barnettii* strains. In order to detect potential synteny block inversions, a dot plot based on MUMmer matches between the two *K. barnettii* strains CLIB 433 and 1767 was produced (fig. 1*B*). These results confirm a nearly perfect collinearity between the two *K. barnettii* strains, excepting scaffolds 02 in the two assemblies which suggests large-scale chromosomal inversions. Figure 1*C* confronts the organization of these two scaffolds. Four breakpoints were identified. The differences between these two sequences may be explained by two hypothetical inversion events. Analysis of the breakpoints showed that one is at the *MAT* locus and another at a silenced cassette, the *HMR* locus. Validation of these rearrangement events were conducted by performing PCR amplifications on the *MAT* locus of the two strains, and by analyzing the mate-pair read mapping at the different inversion breakpoints. These two analyses are detailed in supplementary file S1, Supplementary Material online.

The structure of the assemblies of the two *K. barnettii* strains is very similar with a particularly high degree of synteny. This offers an excellent opportunity to identify orthologous genes between these two genomes. From the pairwise analysis of the two *K. barnettii* strains with SynChro (minimal size of a synteny block set to 4 genes), and using the InterOrtho.py procedure from the CHROnicle tool suite, with a minimal similarity threshold set to 60%, 5,204 syntenic homologous gene pairs, which can be considered as orthologs, were retrieved.

Based on the analysis performed with SynChro, 5,204 syntenic homologous gene pairs, which can be considered as orthologs, were identified. The manual evaluation of the remaining genes (118 from CLIB 433 and 112 from CLIB 1767) completed the orthologous gene list with 96 additional pairs. The complete list of 5,300 putative orthologous gene pairs between the two *K. barnettii* strains is provided in supplementary table S1, Supplementary Material online. Based on the alignment of the predicted protein products only 21 genes (0.4%) have a pairwise similarity of less than 98% while 4,774 (90.1%) of them are identical.

This comparison also revealed that among the 5,300 putative orthologous gene pairs, 12 have a pseudogene for one of the two strains (see supplementary file S1, Supplementary Material online). There are eight pseudogenes in CLIB 433 whose orthologs are complete in both sourdough strains CLIB 1767 (*K. barnettii*) and CLIB 1764 (*K. saulgeensis*). Three of these have no paralogs in the rest of the genome, implying that their absence corresponds to a loss of function in CLIB 433. Conversely, CLIB 1767 has four pseudogenes that are apparently functional genes in CLIB 433. Only one of them has complete copies elsewhere in the genome.

Lastly, in order to identify possible strain-specific genes in the two *K. barnettii* strains, inspection of the 22 genes from CLIB 433 and the 18 genes from CLIB 1767 with no evidence of orthology relationship was carried out. Of these 40 genes, all but one could be found on the shorter (<10 kb) nonretained scaffolds from the assemblies, or were represented in the unassembled reads, and hence probably resulted from incomplete assembly. The one gene which appeared to be specific (KABA2_13S06644, from the sourdough strain CLIB 1767) was predicted to encode a hypothetical protein of 650 aa length having a putative Beta-mannosyltransferase domain (InterProScan prediction; family IPR021988; Blum et al. 2021).

### Inspection of Mating Type Loci

Ability to switch mating types has not been experimentally tested in *K. barnettii*, but complete copies of the *HO* endonuclease gene are present in the genomes of the both studied strains (KABA1_07S08096 and KABA2_07S08074). Chromosomal rearrangement at the *MAT* locus has previously been observed in *K.africana*. In that particular case, the breakage has led to the separation of the mating type loci between two chromosomes (chromosomes 1 and 4), and to the loss of the silent cassettes *HML* and *HMR*, as well as the *HO* endonuclease gene (Gordon et al. 2011; Wolfe et al. 2015). A detailed analysis of the mating type loci of the *K. barnettii* strains including a comparison with two other *Kazachstania* species *K. saulgeensis* CLIB 1764 and *K. naganishii* CBD 8797 and with *Saccharomycescerevisiae* S288c is available in supplementary file S1, Supplementary Material online.

### Duplicated Actin Gene

The actin encoding gene (*ACT1*) is an essential, ubiquitous, and highly conserved gene in eukaryotic organisms. In yeasts, it is one of the most commonly used marker genes in taxonomic analyses (Daniel et al. 2001; Daniel and Meyer 2003; Stielow et al. 2015). Inspection of the assemblies of the two strains of *K. barnettii* revealed that their genomes contain two copies of the gene encoding actin. It is noteworthy that actin is encoded as a single gene in *S. cerevisiae* and, to our knowledge, while duplicated actin genes have been reported in various other eukaryotic taxa, there is no study reporting such duplications in another Saccharomycotina species. To decipher the origin of this duplication, a synteny analysis around *ACT* loci from several species from *Kazachstania* and other related genera was performed (supplementary file S1, Supplementary Material online). This revealed that the two actin gene regions result from a large duplication (probably the WGD), followed by differential gene loss. The discovery of two genes encoding actin in *K. barnettii* and *K. saulgeensis* suggests that this marker is not appropriate to identify species in the *Kazachstania* clade.

## Materials and Methods

### Biological Materials

CLIB 1767 is a haploid spore (B13-6-E7) of a diploid strain (B13-6) that was isolated in April 2013 from a natural wheat sourdough made by a baker located in the Jura department of France. CLIB 433 is one of the two strains used to describe *K. barnettii* species. It was isolated as a spoilage agent of soft drink, before 1978 and deposited at the CBS under the species name *Saccharomyces exiguus* (CBS 6946, no longer available at the CBS collection) (Yarrow 1978).

### DNA Extraction, Sequencing, and Assembly

Genomic DNA was extracted from the two *K. barnettii* strains CLIB 433 and CLIB 1767 using the Nucleospin Plant II Protocol (Machery-Nagel) adapted for yeast genomic DNA preparation MidiPrep (Jacques et al. 2016). Using the Illumina HiSeq 2500 platform (BGI, China), a mate-pair library of 6-kb insert size was sequenced, generating 6.12 and 9.26 million read pairs of 125 bp for CLIB 433 and CLIB 1767, respectively. This led to a 120× to 180× average coverage. Quality-based trimming was performed using Trimmomatic tool (version 0.32) (Bolger et al. 2014) with the following parameters: ILLUMINACLIP:TruSeq3-PE.fa:2:30:10 LEADING:3 TRAILING:3 SLIDINGWINDOW:4:25 MINLEN:50. The reads were assembled with Platanus (version 1.2.1) (Kajitani et al. 2014) with default parameter values (i.e., *u* = 0.1). GapCloser (version 1.12) (Luo et al. 2012) was then used to fill scaffold gaps. The rDNA unit was assembled separately and manually integrated between the two scaffolds identified as flanking the rDNA after mate-pair read mapping using BWA (Li and Durbin 2009).

### Structural and Functional Annotation

Based on the reference genomes of two well annotated reference strains of related species, *S.cerevisiae* strain S288c (release R64-2-1) and *Lachancea kluyveri* strain NRRL Y-12651 (accession: GCA_000149225.1), the putative protein-coding genes (CDS) were annotated using the Amadea Annotation transfer tool (Isoft, France). Additional putative CDS were added based on BLAST results against the NCBI nonredundant database and from prediction of CDS longer than 150 aa with ORF Finder (Sayers et al. 2011). Transposable elements were identified using the NCBI BLAST+ (Camacho et al. 2009), with known Ty1, Ty3, and hAT sequences as queries. Genes coding for tRNAs were identified using tRNAscan-SE v1.3.1 (Chan and Lowe 2019). Functional annotation was based on protein similarity to *S. cerevisiae*. Annotation of coding sequences with no similarity to those in *S. cerevisiae* was performed by comparison with the REFSEQ database. Manual annotation of the two sequenced genomes was performed in the Orcae database (Sterck et al. 2012).

The BUSCO procedure version 4.0.5 (Simão et al. 2015) was applied in order to evaluate the completeness of the assemblies and the annotations of the two *K. barnettii* strains. The “saccharomycetes_odb10” was considered as the reference data set.

### Genome Comparison and Analysis

Synteny between the genome sequences of the two *K. barnettii* strains (CLIB 433 and CLIB 1767) and of the *K. saulgeensis* strain (CLIB 1764) was first evaluated with SynChro (Drillon et al. 2014) with parameter setting Δ = 3 (i.e., a minimal synteny block must contain at least 4 genes). Then, in order to visualize possible intrachromosomal rearrangements, the MUMmer tool suite (version 4.0.0rc1) (Marçais et al. 2018) was used.

Orthologous groups of proteins were reconstructed with the command InterOrtho.py of SynChro (Drillon et al. 2014) with a minimal pairwise protein similarity set to 60%. The advantage of this tool is that it considers both protein homology and synteny to build orthologous relationships. SynChro was run on the proteomes of the two *K. barnettii* strains (CLIB 433 and CLIB 1767) and of *K. saulgeensis* strain CLIB 1764 with parameter setting Δ = 3. In order to reduce complexity and to avoid possible errors due to mobile elements, transposon-derived sequences were excluded from this analysis.

To complete the list of orthologous groups provided by SynChro when comparing the two *K. barnettii* strains, a subsequent analysis including the genes rejected by SynChro as well as all the pseudogenes of the two strains was conducted. Indeed, SynChro does not consider pseudogenes in orthologous group reconstruction. In addition, several proteins can be excluded from the analysis due to a high copy number or proximity of assembly gaps. Lastly, although the proposed assemblies are almost complete, a few genes may be missing from the retained scaffolds but found in the full set of assembly contigs. This procedure was performed with BLASTp and tBLASTn tools from the NCBI BLAST+ tool suite (version 2.10.0+) (Camacho et al. 2009) and the obtained orthologous protein pairs were validated on the basis of synteny provided by SynChro.

Last, identity and similarity of each orthologous coding gene pair were evaluated at the protein and the nucleotide levels from a pairwise global alignment performed with the Needleman–Wunsch algorithm (protein similarities were computed with BLOSUM62 matrix) (Rice et al. 2000). 

## Supplementary Material

Supplementary data are available at *Genome Biology and Evolution* online.

## Supplementary Material

evac007_Supplementary_Data
