# Supplementary material for: Whole-Genome Sequences of Two Kazachstania barnettii Strains Isolated from Anthropic Environments
Source: Genome Biol Evol. 2022 Feb 1;14(2):evac007. doi: 10.1093/gbe/evac007 (PMC8825440; doi:10.1093/gbe/evac007)
Supplement: evac007_Supplementary_Data [file evac007_supplementary_data.zip › R1_Supplementary_file_S1.docx]

## Inversion breakpoint validation on scaffold 02

The only genome structure variations identified between strains CLIB 433 and CLIB 1767 are the chromosomal inversions in scaffold 02 of the two assemblies. Four breakpoints were identified. The differences between these two sequences can be explained by two hypothetical inversion events (see **Figure 1C** of the manuscript). Analysis of the breakpoints showed that one is at the *MAT* locus and another at a silenced cassette, the *HMR* locus. To ensure that these inversions are not assembly artifacts, investigation of the mate-pair mapping was performed at the four breakpoints using BWA MEM tool (version 0.7.17) (Li and Durbin, 2009). Thus, when mapping CLIB 1767 mate-pair reads against the CLIB 433 sequences, clear coverage interruptions happen at the four breakpoints, while no interruption can be observed when considering reads of CLIB 433 against itself (see **Figure S1**).


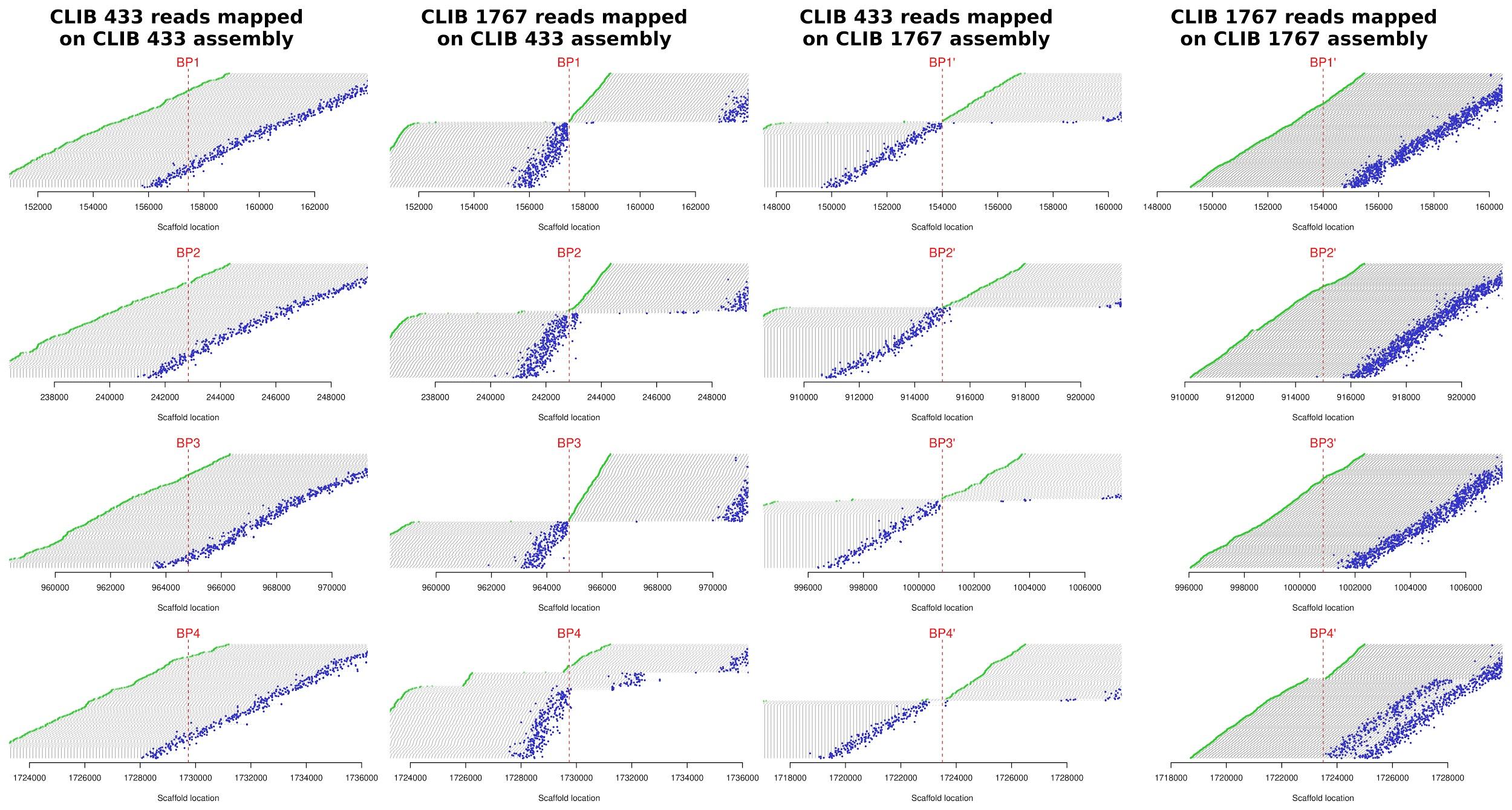


**Figure S1**: Mate-pair mapping on the four identified breakpoints (BP1 to BP4 on CLIB 433 scaffold, and BP1’ to BP4’ on CLIB 1767 scaffold). Read pairs covering these regions are represented by a couple of dots, one green for the left member and one blue for the right member, connected by a horizontal dashed grey line. The red columns correspond to the mapping of CLIB 433 reads and the green ones to the CLIB 1767 reads. The two first columns consider the CLIB 433 assembly as reference and the two last ones consider the CLIB 1767 assembly as reference.

## PCR analysis of the *MAT* loci

Three primers were designed from the genome assemblies.

>pA

AGTTGAATTGGGCCGCTTTT

Intergenic, (near 5’ end of KABA1_02S02200 and KABA2_02S16280, similar to Torulaspora delbrueckii TDEL_0C05700)

>pB

GGCTGCCCCAATTGTTAGAG

Intragenic (in KABA1_02S16346 and KABA2_02S08976, similar to *Saccharomyces cerevisiae* YMR315W)

>pC

GCGTTTTGACCACCAGCTGAA

Intragenic (in KABA1_02S16434 and KABA2_02S16324, similar to *Saccharomyces cerevisiae* CAN1)


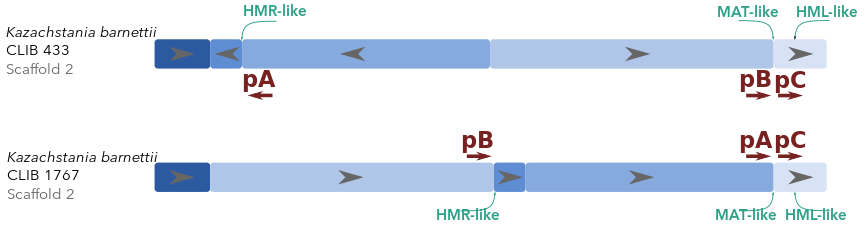


**Figure S2**: Position of the three primers on the scaffold 02 of the two assemblies.

Nucleic acids were extracted following the procedures of Hoffman and Winston 1987.

Two PCRs were performed, one with the couple of primers A and C and another between B and C (see **Figure S2**). The two sequences were PCR-amplified with the Expand™ Long Template PCR System (Roche) in a final reaction mixture of 50µl containing between 25 and 50 ng of genomic DNA, 0.8 mM dNTPs, 0.4 µM forward and reverse primers in the buffer 1 and 1 U Taq. Amplification reactions were run on a 2720 thermal cycler (Applied Biosystems) as follows: 2 min at 94°C, followed by 25 cycles of 10 s at 94°C, 190 s at the annealing temperature (55 °C for the couple A/C, 60 °C for the couple B/C) and 90 s at 68°C, with a final extension step of 7 min at 68°C. PCR products were separated by electrophoresis using a 1% agarose gel.

The resulting amplicons were sequenced on both strands by Eurofins MWG Operon (Ebersberg, Germany). Sequencing primers were those used for PCR amplification. Sequences were assembled with the phred/phrap/consed package (Ewing and Green 1998).

In CLIB 433, an amplification is obtained only when considering primers pB and pC:

>CLIB_433_pBpC

TTATTTCTTTGAAATCATTGTATATGTGTTTTGTTTATTGAATTTTCGAAGGAGATTTTT

CCGTTTTAGGAAATACGGAATTACATCAAAAAATTAACGTCATAAAAAATCTCGTTTTTT

CTTCATTCTTGCATTTGAAAAGTAAACAAATTTCGTAAACAAATCAAAAATTAGACTTTA

TAACCTTCAAATTGATTCCTTTAATCCCATTTTCTATGCTTTCCATCAATTTTGATCAAG

GACATCTTGCTTTTAGGGACATTTCTTGATTCGGGACTTAGTTTGCATGAAAAGAAGGAA

CTTTTAAAAAGGTTGTACGATAACTACAACCTTCTGGTTGTACCAAAGAAACGTAAGAGA

ACCACTATTAGTAAGAGTACTAAAGAATTTTTGGAAAAAGTATTTGAAAAGAAACAGTGG

ATCACCAGGGAAGAAAGACAGATTGTGGCAATGGAGTGTGGTATCACACCATTACAAGTC

AGAATATGGGTATGTTGTTACTTGTATACATTTACTACTCATCATTACATTTTACTAACA

ATATCATATATATATATATATATATATATAATGTAGTTTATAAACAAACGATCACGGACT

AAACCGTCAGTGTCTAATACATAGTACATATAATTATGATCACATCAATGCAGGAGTTTT

GTAGAAAGAAATGAATATAGAGAGGGTCTAAGAGGGATTCCATATGAGTGCGTCTAAGTG

TCTTCTGCCTGAGTCTAGGTCCAGTTGGTGGAGCGGGAGAAGGATAGGCGAGCGGAAGTA

GAGCTGGAACGCAATGTAAAGGCCTATGAAAAGGAATATGGATATATAGGCTGCGAAAAA

ATCAGATGTAGAAAACTGAGGTGCGAACGCAGTAAACCCTTGAAGAATAATGATCAGAAT

CATGAAAAATGTCGCATAATAGGCTAACCCAGGCATTAGTGAAGCTTTGAATGGAAGGTC

ATTTCGTGATATATTACGGTAACGGAGAGCCTTCATGAATCGAATATGGGCCAATGAAAT

GAATAACCATGAAAAGAAACCTGCTACTGCG

In CLIB 1767, an amplification is obtained only when considering primers pA and pC:

>CLIB_1767_pApC

CATATTTTGTTTTATAATCGTTTTAGAAGGCTTAAAAATTAAAGAAAAATAAAATAAGTA

TAGTTAAAAAAATGAATCAAAATATATAAAGACCTTAAAGGAGTAAGTTACAGACGCAAA

AGTTTTTGAAAAAAGGTAAACAAAAAGAGCAAACAAATAAGGATCAGATGAATATAGATA

TTGTGCAACAGAGAGATTTCAATTATATTAGCAAAGTATGTTAATTTGGGAAAATATAGA

AGGGCGAGAATAAGTATTACTAACACTGTAGTAGGATATCCTTGATTCGGGACTTAGTTT

GCATGAAAAGAAGGAACTTTTAAAAAGGTTGTACGATAACTACAACCTTCTGGTTGTACC

AAAGAAACGTAAGAGAACCACTATTAGTAAGAGTACTAAAGAATTTTTGGAAAAAGTATT

TGAAAAGAAACAGTGGATCACCAGGGAAGAAAGACAGATTGTGGCAATGGAGTGTGGTAT

CACACCATTACAAGTCAGAATATGGGTATGTTGTTACTTGTATACATTTACTACTCATCA

TTACATTTTACTAACAATATCATATATATATATATATATATATATAATGTAGTTTATAAA

CAAACGATCACGGACTAAACCGTCAGTGTCTAATACATAGTACATATAATTATGATCACA

TCAATGCAGGAGTTTTGTAGAAAGAAATGAATATAGAGAGGGTCTAAGAGGGATTCCATA

TGAGTGCGTCTAAGTGTCTTCTGCCTGAGTCTAGGTCCAGTTGGTGGAGCGGGAGAAGGA

TAGGCGAGCGGAAGTAGAGCTGGAACGCAATGTAAAGGCCTATGAAAAGGAATATGGATA

TATAGGCTGCGAAAAAATCAGATGTAGAAAACTGAGGTGCGAACGCAGTAAACCCTTGAA

GAATAATGATCAGAATCATGAAAAATGTCGCATAATAGGCTAACCCAGGCATTAGTGAAG

CTTTGAATGGAAGGTCATTTCGTGATATATTACGGTAACGGAGAGCCTTCATGAATCGAA

TATGGGCCAATGAAATGAATAACCAGAAAAGAAACC

## List of pseudogene in one of the two *K. barnettii* strains

Among the 5300 putative orthologous gene paires, 12 have a pseudogene for one of the two strains (see **Table S2**). This pseudogene count does not include uncompleted genes disrupted by scaffold ends or assembly gaps, and hence they represent possible function losses. For comparison purposes, orthologous genes from K. saulgeensis CLIB 1764 are also presented in **Table S2**. There are 8 pseudogenes in CLIB 433 whose orthologues are complete in the both sourdough strains CLIB 1767 (*K. barnettii*) and CLIB 1764 (K. saulgeensis). Among these, 3 have no paralogues in the rest of the genome, implying that their absence will correspond to a loss of function in CLIB 433 (*K. barnettii*, reference). Concerning CLIB 1767, 4 pseudogenes are present as apparently functional genes in CLIB 433 and one is a pseudogene in CLIB 1764. Only one of them has complete copies elsewhere in the genome.

**Table S2:** Orthologous gene pairs that include a functional copy of the gene and a pseudogene between the two *K. barnettii* strains CLIB 433 (reference) and CLIB 1767 (sourdough) and the *K. saulgeensis* strain CLIB 1764 (sourdough). Pseudogenes are indicated in bold face.

| **CLIB 433** | **CLIB 1767** | **CLIB 1764** | **Unique^1^** | **Annotations^2^** |
| --- | --- | --- | --- | --- |
| **KABA1_01S02398** | KABA2_01S02398 | KASA_0Q04334G | Y | GZF3 |
| **KABA1_01S16830** | KABA2_01S16764 | KASA_0Q11814G | N | KSS1 |
| **KABA1_01S20240** | KABA2_13S02794 | KASA_0Q13552G | N | NDE2 |
| **KABA1_03S03058** | KABA2_03S03058 | KASA_0O01628G | N | KTR4 |
| **KABA1_03S03410** | KABA2_03S03410 | KASA_0O01804G | Y | CAD1 |
| **KABA1_05S10582** | KABA2_05S10626 | KASA_0E00121G | N | RDS1 |
| **KABA1_06S02640** | KABA2_06S02684 | KASA_0K01474G | Y | LDB19 |
| **KABA1_11S02464** | KABA2_11S02420 | KASA_0G01089G | N | OYE2 |
| KABA1_02S01408 | **KABA2_02S01386** | KASA_0I00671G | Y | Hypoth. prot. |
| KABA1_03S01738 | **KABA2_03S01738** | KASA_0O00913G | Y | HAL9 |
| KABA1_04S12738 | **KABA2_04S13046** | **KASA_0P06655G** | N | MNN4 |
| KABA1_08S07128 | **KABA2_08S05896** | KASA_0F01507G | Y | YEN1 |

1. Indicates whether the pseudogene is the unique copy of the gene in the genome (Y) or if there exists at least one functional copy of an homologous gene in the rest of the genome (N) (e.g., paralogous genes, multigenic families).
2. Similar/homologous genes in the annotation of *S. cerevisiae* S288c.

## Organisation of the mating type loci

The mating type loci of the two *K. barnettii* strains were compared with those of three other species: *K. saulgeensis* CLIB 1764, *K. naganishii* CBS 8797 and *S. cerevisiae* S288c. Results are presented on **Figure S3**, which schematizes the organization of the mating type loci and the immediate flanking genes. The *HML* region, at the right of the figure, is conserved in all the five species while *HMR* is only conserved between *K. barnettii* and *K. saulgeensis*. Mating type locus organizations of *K. barnettii* CLIB 1767 and *K. saulgeensis* CLIB 1764 are broadly similar to that of *K. naganishii* CBD 8797 (Wolfe *et al.* 2015) except for the *HMR* locus environment which is different and more distant from the *MAT* locus. Structure of *K. barnettii* CLIB 433 mating type loci confirms the putative inversion events hypothesized in the previous section. Furthermore, these broader comparisons add weight to the hypothesis that these inversions occurred in a recent ancestor of CLIB 433.


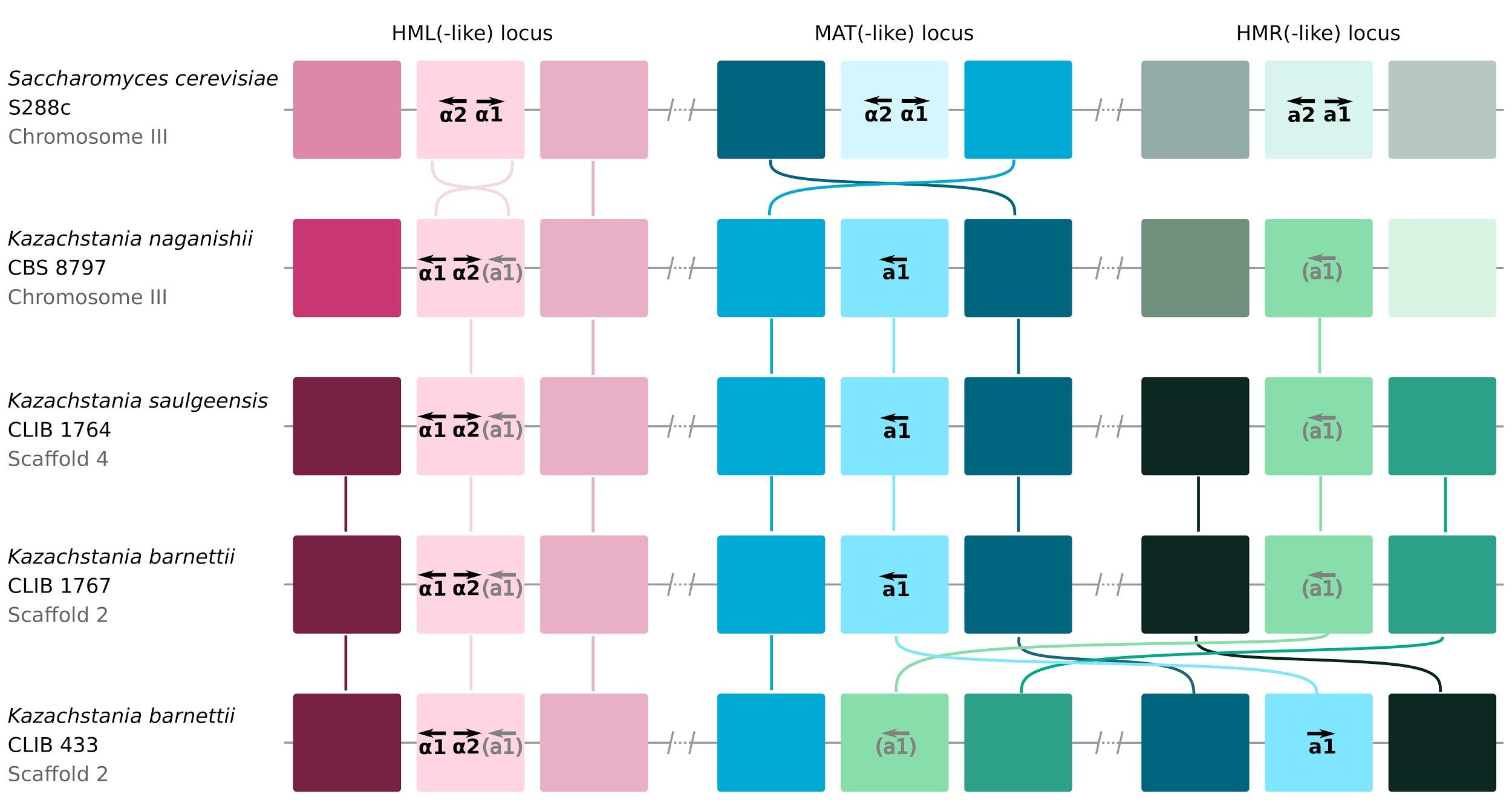


**Figure S3**: Organization of mating mating type loci for *S. cerevisiae* S288c, *K. naganishii* CBS 8797, *K. saulgeensis* CLIB 1764, and the *K. barnettii* strain CLIB 1767 and CLIB 433. Organisation of each locus is represented by three colored boxes, the central one contains the mating type genes (*i.e.*, a1, alpha 1 and alpha 2) and the two surrounding ones represent flanking genes. Blocks of the same color between species and connected by a line represent conserved regions with shared syntenic genes. Black arrows represent the strand orientation of genes and pseudogenes are shown in brackets and in grey.

## Duplicated Actin genes

The actin encoding gene (ACT1) is an essential, ubiquitous and highly conserved gene in eukaryotic organisms. In yeasts, it is one of the most commonly used marker genes in taxonomic analyses (Daniel, Sorrell and Meyer 2001; Daniel and Meyer 2003; Stielow et al. 2015). Inspection of the assemblies of the two strains of *K. barnettii* revealed that their genomes contain two copies of the gene encoding actin. It is noteworthy that actin is encoded as a single gene in *S. cerevisiae* and, to our knowledge, while duplicated actin genes have been reported in various other eukaryotic taxa, there is no study reporting such duplications in another Saccharomycotina species.

The first copy of the actin gene, which we designate ACT1, is located on the first scaffold of the two *K. barnettii* strains. An assembly gap crops the 3’ end of the gene in CLIB 433, with about 40 missing bases. The two genes are identical at the protein level, they contain 4 silent substitutions in their coding regions (CDS) and their introns are identical. A second copy (ACT2) of the actin gene is located on the third scaffold of the two assemblies. These orthologous genes are identical at both the nucleotide and the protein levels. There are 10, silent nucleotide differences between the paralogous genes in CLIB 433 and 9 in CLIB 1767.

Inspection of the *K. saulgeensis* genome also revealed two copies of the actin encoding gene, one complete copy on scaffold 03 (KASA_0O07095G), and on partial copy on scaffold 01 (KASA_0Q10725G), interrupted by an assembly gap. These two ACT loci are syntenic orthologs of the ACT loci found in *K. barnettii* genomes.


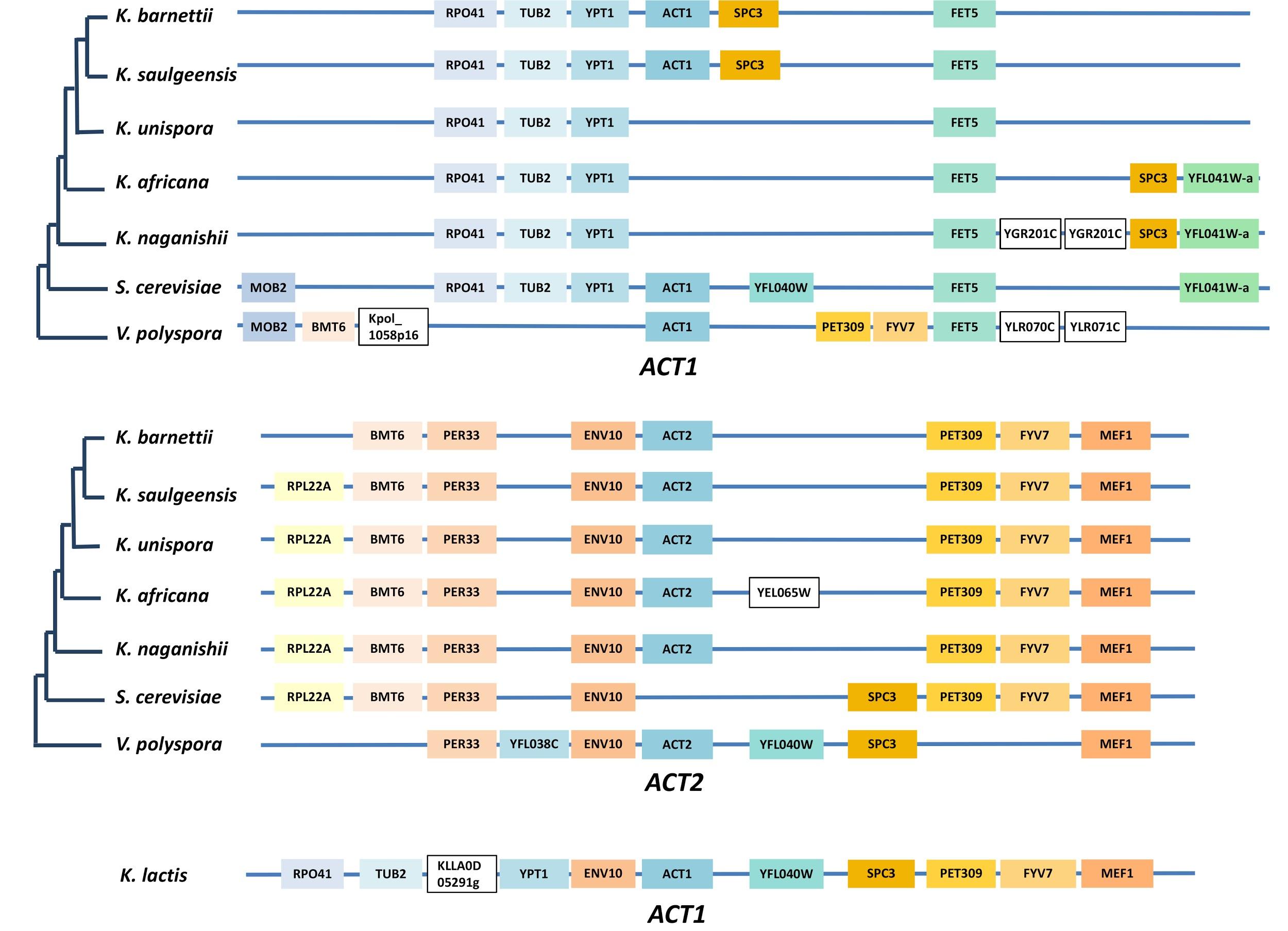


**Figure S4**: Genetic environments of the two actin genes in *K. barnettii* and the corresponding regions in selected *Saccharomycetaceae* genomes.

In order to investigate the origin of this duplication, genes coding for actin were searched for among the other genomes available in the genus *Kazachstania* and in other related genera. This led to the finding that one other species possesses two copies of the gene encoding actin: *Vanderwaltozyma polyspora*. On the other hand, *K. naganishii*, *K. africana* and *K. unispora* contain only one copy of the ACT1 gene. The genomic environment of the detected actin genes was compared for the five *Kazachstania* species (*K. saulgeensis*, *K. barnettii*, *K. unispora*, *K. naganishii*, *K. africana*), two other post-WGD species (*V. polyspora* and *S. cerevisiae*) and the pre-WGD species *Kluyveromyces lactis* (**Figure S4**). Arbitrarily, the actin gene copy from the scaffold 01 of *K. barnettii* and *K. saulgeensis* assemblies was denoted ACT1, while the copy from scaffold 03 was named ACT2. The corresponding genes from the other compared species are also designated respectively ACT1 and ACT2, for the purposes of the **Figure S4**. This comparison shows firstly that the paralogues are located in two different regions in the species containing two ACT genes, indicating that the presence of these genes is not the result of a simple tandem duplication. They are located on the homologs of chromosomes 6 and 12 of *S. cerevisiae*. Observation of the gene organization in different species leads to the conclusion that *S. cerevisiae* and those *Kazachstania* with only one actin gene have retained different copies. Indeed, if we take the disposition in the pre-WGD K. lactis as the ancestral state, it appears that the two actin gene regions result from a large duplication (probably the WGD), followed by differential gene loss. Alternatively, a duplication of the actin gene may have occurred, but this is unlikely since the position of ACT2 is identical in *V. polyspora*, which diverged before the Saccharomyces and the *Kazachstania*. Hence, S. cerevisiae has its single copy ACT1 gene on chromosome 6, whereas in all post-WGD *Kazachstania* species analyzed, as well as in *V. polyspora*, the ACT2 gene was conserved on the equivalent of the *S. cerevisiae* chromosome 12.

The discovery of two genes encoding actin in *K. barnettii* and *K. saulgeensis* reveals that this marker is not appropriate to identify species in the *Kazachstania* clade. The primers used for this marker are generally located inside the coding region of the actin gene, which will lead to the amplification of the two copies. In addition, the upper threshold used to differentiate two strains from the same species on the basis of actin is 10 nt (Daniel and Meyer 2003) while the difference found in this study between the two actin gene copies is also 10 nt.

## References

Daniel H-M, Meyer W. Evaluation of ribosomal RNA and actin gene sequences for the identification of ascomycetous yeasts. *Int J Food Microbiol* 2003;**86**:61–78.

Daniel HM, Sorrell TC, Meyer W. Partial sequence analysis of the actin gene and its potential for studying the phylogeny of *Candida* species and their teleomorphs. *Int J Syst Evol Microbiol* 2001;**51**:1593–606.

Ewing B, Green P. Base-calling of automated sequencer traces using phred. II. Error probabilities. *Genome Res* 1998;**8**:186–94.

Hoffman CS, Winston F. A ten-minute DNA preparation from yeast efficiently releases autonomous plasmids for transformation of Escherichia coli. *Gene* 1987;**57**:267–72.

Li H, Durbin R. Fast and accurate short read alignment with Burrows–Wheeler transform. *Bioinformatics* 2009;**25**:1754–60.

Stielow JB, Lévesque CA, Seifert KA *et al.* One fungus, which genes? Development and assessment of universal primers for potential secondary fungal DNA barcodes. *Persoonia Mol Phylogeny Evol Fungi* 2015;**35**:242–63.

Wolfe KH, Armisén D, Proux-Wera E *et al.* Clade- and species-specific features of genome evolution in the *Saccharomycetaceae*. *FEMS Yeast Res* 2015;**15**:fov035.
